# Transcriptomic Profiling Identifies TALAM1 and LINC00702 as HIV-1-Responsive lncRNAs in Microglia

**DOI:** 10.3390/ijms27073271

**Published:** 2026-04-04

**Authors:** Victoria Rojas-Celis, Catalina Millan-Hidalgo, Izabela Mamede, Isidora Morales-Vejar, Isidora Pittet-Díaz, Diego Fernández-Rodríguez, Paulo P. Amaral, Helder I. Nakaya, Sebastián Reyes-Cerpa, Fernando Valiente-Echeverría, Camila Pereira-Montecinos, Daniela Toro-Ascuy

**Affiliations:** 1 Virology Laboratory, Department of Biology, Faculty of Sciences, Universidad de Chile, Santiago 7800003, Chile; victoria.rojcel@gmail.com (V.R.-C.); ctln.millanhidalgo@gmail.com (C.M.-H.); isidora.morales.v@ug.uchile.cl (I.M.-V.); isidora.pittet@gmail.com (I.P.-D.); 2 Laboratory of Genetics Biochemistry, Departamento de Bioquímica e Imunologia, Instituto de Ciências Biológicas, Universidade Federal de Minas Gerais, Belo Horizonte 31270, Brazil; iza.mamede@gmail.com; 3 Escuela de Nutrición y Dietética, Facultad de Medicina y Ciencias de la Salud, Universidad Mayor, Santiago 8580745, Chile; diego.fernandezr@umayor.cl; 4 Centro de Genómica y Bioinformática, Facultad de Ciencias, Ingeniería y Tecnología, Universidad Mayor, Santiago 8580745, Chile; sebastian.reyes@umayor.cl; 5 INSPER-Institute of Education and Research, São Paulo 04546, Brazil; paulopra@insper.edu.br; 6 Hospital Israelita Albert Einstein, São Paulo 05620, Brazil; hnakaya@usp.br; 7 Department of Clinical and Toxicological Analyses, School of Pharmaceutical Sciences, University of São Paulo, São Paulo 05508, Brazil; 8 Escuela de Biotecnología, Facultad de Ciencias, Ingeniería y Tecnología, Universidad Mayor, Santiago 8580745, Chile; 9 Molecular and Cellular Virology Laboratory, Núcleo Interdisciplinario de Microbiología, Instituto de Ciencias Biomédicas (ICBM), Center for HIV/AIDS Integral Research (CHAIR) and Instituto Milenio en Inmunología e Inmunoterapia, Facultad de Medicina, Universidad de Chile, Santiago 8380453, Chile; fvaliente@uchile.cl

**Keywords:** lncRNAs, HIV-1, TNF-α, microglia, TALAM1, LINC00702

## Abstract

Microglia, the resident macrophages of the central nervous system (CNS), serve as the primary reservoir of HIV-1 in the brain and play a crucial role in the development of HIV-1-associated neurocognitive disorders (HAND). While long non-coding RNAs (lncRNAs) have emerged as essential regulators of HIV-1 replication in T cells and macrophages, their role in microglia remains poorly understood. Here, we performed RNA sequencing of polyadenylated transcripts from a human microglial cell line exposed to HIV-1 infection or TNF-α stimulation to investigate transcriptional responses and identify lncRNAs with potential regulatory functions. Gene set enrichment analysis revealed broad overlap between viral and inflammatory responses, reflecting convergence on common molecular pathways. Among differentially expressed lncRNAs, we focused on TALAM1, which was specifically induced by HIV-1, and LINC00702, which responded to both HIV-1 and TNF-α. Validation by RT-qPCR confirmed the upregulation of TALAM1 and LINC00702 at 24 h post-infection. Furthermore, knockdown of either lncRNA affected viral genomic RNA levels, while only LINC00702 knockdown affected p55 production. Given that subcellular localization informs lncRNA function, we assessed the distribution of TALAM1 and LINC00702. TALAM1 was predominantly cytoplasmic under basal conditions but shifted toward nuclear enrichment upon HIV-1 infection, whereas LINC00702 remained primarily nuclear regardless of infection status. Consistent with their genomic context, protein interaction predictions, and pathway enrichment analyses suggested that TALAM1 may influence RNA processing and splicing, whereas LINC00702 may contribute to translational regulation and is associated with proteins involved in immune responses. Together, these findings provide an initial characterization of lncRNA responses to HIV-1 infection in a human microglial cell line and identify TALAM1 and LINC00702 as candidates for future functional studies in the context of viral infection and neuroinflammation.

## 1. Introduction

Microglia are the resident macrophages of the central nervous system (CNS) and represent the main reservoir of human immunodeficiency virus type 1 (HIV-1) in the brain [1,2]. Following infection, HIV-1 integrates preferentially into transcriptionally active genomic regions of microglia [3]. This integration pattern facilitates the recruitment and activation of transcription factors such as nuclear factor κB (NF-κB), signal transducer and activator of transcription 1 (STAT1), and activator protein 1 (AP-1), promoting viral gene expression and amplifying host inflammatory signaling [3]. Consequently, infected microglia exhibit a robust proinflammatory response characterized by the release of cytokines, including tumor necrosis factor-alpha (TNF-α), interleukin-1 beta (IL-1β), interleukin-6 (IL-6), and interleukin-8 (IL-8), along with the induction of interferon-stimulated genes (ISGs) and repression of cell cycle regulators such as E2F targets and G2M checkpoint genes [4,5,6,7]. This proinflammatory environment, together with viral proteins such as Tat, gp120, Vpr, and Nef released by infected microglia, exerts neurotoxic effects that contribute to the development of HIV-1-associated neurocognitive disorders (HAND) [8]. While a combination of antiretroviral therapy (cART) effectively suppresses viral replication, it does not eliminate latent reservoirs, whereby 15–55% of treated individuals still develop some form of HAND [9].

Recent advances in transcriptomic technologies have identified numerous molecules with therapeutic potential, including long non-coding RNAs (lncRNAs) [10]. These transcripts are typically longer than 200 nucleotides and lack coding potential. They form secondary structures that enable interactions with DNA, RNA, microRNAs, proteins, and other lncRNAs [11]. Through such interactions, they regulate diverse cellular processes, including chromatin remodeling, transcriptional control, RNA splicing, miRNA degradation, and translation [12]. In addition, lncRNAs can localize to distinct cellular compartments, including the nucleus and cytoplasm, and their subcellular distribution is often associated with their regulatory mechanisms [13]. Interestingly, more than 40% of known lncRNAs are expressed in different areas of the brain [14]. In microglia, increasing evidence indicates that their activation state and inflammatory functions are tightly regulated by lncRNAs under various pathological conditions [15]. For example, lncRNA AK148321 can attenuate microglial neuroinflammation [16], whereas MALAT1 overexpression promotes the production of proinflammatory cytokines in lipopolysaccharide (LPS)-treated microglial cells [17]. The lincRNA-p21 contributes to microglial activation and inflammatory signaling [18]. Similarly, lncRNA TUG1 mediates microglial inflammatory activation by regulating glucose metabolic reprogramming [19]. In the context of viral infections, linc-AhRA has been shown to negatively regulate the innate antiviral response in murine microglia upon infection with neurotropic herpesviruses [20]. Regarding HIV-1, accumulating evidence suggests that infection alters the expression of numerous lncRNAs, particularly in T cells and macrophages [21]. However, differential expression alone does not necessarily imply functional relevance, and experimental approaches such as loss-of-function or knockdown strategies are often required to determine whether specific lncRNAs actively contribute to viral replication or host cellular responses. Consistent with this, several lncRNAs have been functionally implicated in the regulation of HIV-1 transcription, such as uc002yug.2 [22], HEAL [23], MALAT1 [24], and CYTOR [25], while others repress it, including NRON [26], 7SK [27], GAS5 [28], AK13018 [29], NKILA [30], and Tar-gag [31]. In addition, lncRNAs such as NEAT1 [32] regulate HIV-1 post-transcriptionally, whereas SAF [33] and lincRNA-p21 [34] promote apoptosis of infected cells, and CCR5AS modulates viral entry by regulating coreceptor expression [35]. Despite this progress, most insights have come from studies on T cells or macrophages, and the contribution of lncRNAs to HIV-1 infection in microglia remains poorly understood. HEAL is the only lncRNA whose mechanism has been characterized in detail in microglia; it modulates HIV-1 transcription through interaction with the RNA-binding protein FUS, underscoring the potential of lncRNAs to regulate viral gene expression directly [23]. Other lncRNAs, including NEAT1 and ZBTB11-AS1, show altered expression across different stages of HIV-1 infection in microglia and correlate with inflammatory responses [36], although their specific mechanisms and roles in the viral replication cycle remain unclear. Since microglia are highly permissive to HIV-1 and can proliferate while carrying the virus [37,38], elucidating their lncRNA-mediated responses may reveal mechanisms relevant to CNS-targeted therapeutic strategies. In this study, we investigated transcriptomic changes in a human microglia cell line following HIV-1 infection and TNF-α stimulation, with a focus on lncRNAs that may contribute to viral or inflammatory responses triggered by the virus. We identified two lncRNAs of interest: TALAM1, uniquely upregulated by HIV-1, and LINC00702, induced under both HIV-1 and TNF-α conditions. We further evaluated their expression, subcellular localization, and impact on viral replication, providing initial insights into their roles in microglial HIV-1 infection, and consistent with their genomic context, protein interaction predictions, and pathway enrichment analyses, TALAM1 may regulate HIV-1 replication by modulating nuclear RNA processing and/or splicing, whereas LINC00702 can influence at the nuclear level in transcriptional processes of HIV gRNA and possibly at the cytoplasmic level with viral protein production through cytoplasmic control of translation. Together, these observations identify previously uncharacterized lncRNAs as candidates for further functional studies in the context of HIV-1 neuropathogenesis and CNS viral persistence.

## 2. Results

### 2.1. HIV-1 Infection and TNF-α Stimulation Alter the Polyadenylated Transcriptome of C20 Human Microglial Cells

**Figure 1:**
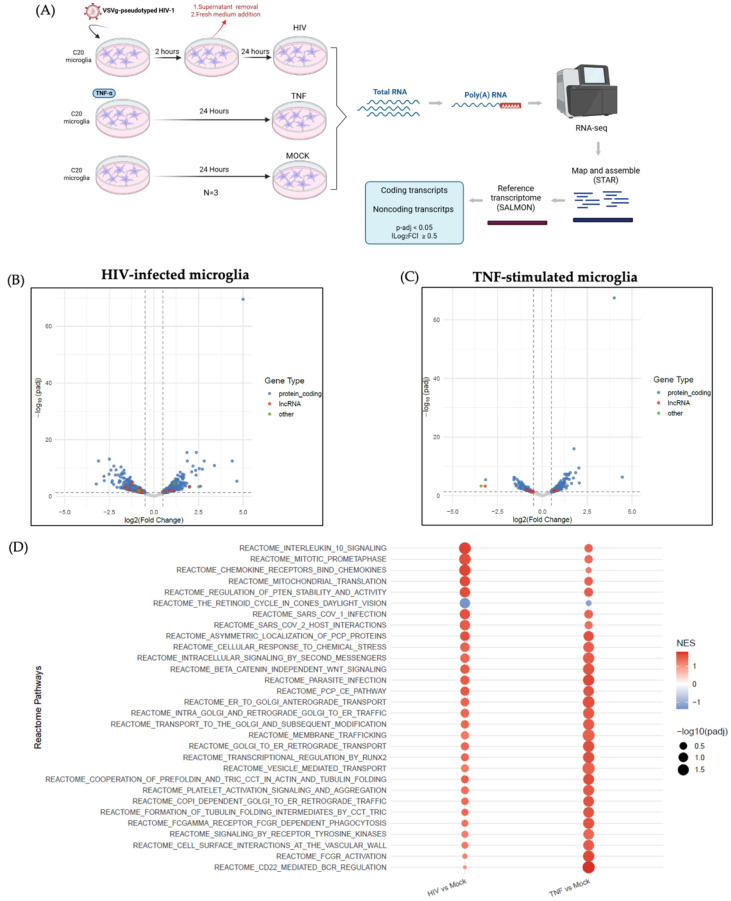
Polyadenylated transcriptome changes in C20 microglial cells upon HIV-1 infection or TNF-α stimulation compared to mock. (**A**) Experimental workflow for RNA-seq analysis of C20 cells under three conditions: untreated (Mock), HIV-1-infected (HIV, 24 h post-infection), and TNF-α-stimulated (TNF, 24 h). Polyadenylated RNA was then sequenced, aligned, quantified, and classified into protein-coding, lncRNA, and other RNA types. (**B**) Volcano plot showing differential expression of protein-coding and non-coding transcripts in HIV compared to Mock. (**C**) Volcano plot showing differential expression of protein-coding and non-coding transcripts in TNF compared to Mock. (**D**) Gene set enrichment analysis (GSEA) of pathways enriched among differentially expressed transcripts in HIV and TNF conditions. Shared pathways include immune response, inflammation, vesicular trafficking, cell cycle regulation, intracellular signaling, and response to viral infection, highlighting common transcriptional programs activated by viral infection and proinflammatory stimulation. Created in BioRender. Toro-Ascuy, D. (2026) https://BioRender.com/qeljpe0 (accessed on 2 December 2025).

To evaluate transcriptomic changes associated with HIV-1 infection and proinflammatory stimulation by TNF-α in microglia, we performed RNA sequencing of polyadenylated transcripts from the human microglial cell line C20 under three conditions: HIV-1 infection, TNF-α stimulation, and untreated controls. For HIV-1 infection, cells were exposed to 250 ng/mL of p24 from VSVg-pseudotyped HIV-1 for 2 h. Afterwards, the viral supernatant was removed and cells were incubated with fresh medium for an additional 24 h. For TNF-α stimulation, cells were treated with 10 pg/mL recombinant TNF-α for 24 h as previously described by Garcia-Mesa et al. (2017) [39]. For simplicity, these conditions are hereafter referred to as HIV, TNF, and Mock, respectively. Successful infection was confirmed by detection of the viral proteins Pr55(Gag) and p24 through Western blot (Supplementary Figure S1A). Lactate dehydrogenase (LDH) release assays indicated that neither HIV-1 infection nor TNF-α stimulation significantly affected C20 microglial cell viability under the experimental conditions used (Supplementary Figure S1B). Polyadenylated RNA was purified from total RNA and subjected to RNA-seq. Sequencing data were processed through a pipeline including transcript alignment (STAR), quantification against a reference transcriptome (SALMON), and classification into coding and non-coding polyadenylated transcripts (Figure 1A). Differential expression was defined as an absolute log_2_(fold change) ≥ 0.5 with an adjusted *p*-value < 0.05 (Figure 1A). A detailed overview of the transcript filtering and prioritization workflow, including the number of transcripts retained at each step, is provided in Supplementary Figure S2A. Principal component analysis (PCA) revealed that experimental conditions accounted for the largest source of variance (PC1 = 54.6%), indicating clear transcriptional separation between groups (Supplementary Figure S2B). As expected from the polyadenylated RNA population, most of the identified transcripts that changed their expression corresponded to protein-coding transcripts, while a smaller fraction corresponded to lncRNAs and other RNAs, both in the infected condition compared to Mock (Figure 1B) and in TNF-α-stimulated cells compared to Mock (Figure 1C). In addition, both TNF-α stimulation and HIV-1 infection induced the upregulation of genes associated with TNF/NF-κB signaling (e.g., NFKB1, RELB, IRAK1) and inflammatory cytokine responses (e.g., IL6, CXCL8), further supporting the biological validity of our experimental approach (Supplementary Table S1). To gain insight into the biological pathways associated with these transcripts that changed their expression, we performed a Gene Set Enrichment Analysis (GSEA), which calculates the Normalized Enrichment Score (NES) to assess both the magnitude and direction of pathway enrichment. The analysis revealed multiple Reactome pathways significantly enriched in both HIV and TNF conditions relative to Mock, including those related to immune response, inflammation, vesicular trafficking, cell cycle regulation, intracellular signaling, and response to viral infection (Figure 1D). However, the differences in NES values between conditions suggest that the magnitude and direction of pathway activation may differ between viral infection and cytokine stimulation. Immune-related pathways showed stronger enrichment in HIV, while vesicle transport or cellular homeostasis processes exhibit relatively higher NES values in TNF-stimulated cells. Together, these results validate our model by confirming that both conditions induce the expected transcriptional signatures and demonstrate that we captured changes not only in protein-coding transcripts but also in lncRNAs.

### 2.2. HIV-1–Specific Responses and TNF-α–Related Inflammatory Programs in Microglia

**Figure 2:**
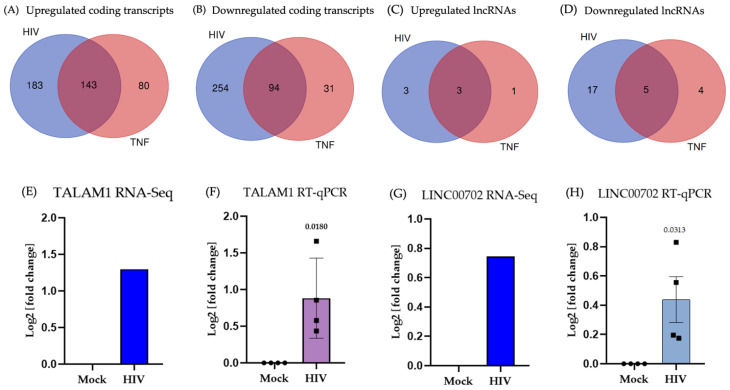
Distinct transcriptional programs triggered by direct viral infection versus TNF-α-mediated inflammatory signaling. (**A**) Overlap of upregulated protein-coding genes in HIV-infected (HIV) and TNF-α-stimulated (TNF) microglia compared with untreated controls (Mock). (**B**) Overlap of downregulated protein-coding genes in HIV and TNF conditions. (**C**) Overlap of upregulated long non-coding RNAs (lncRNAs) in HIV and TNF. (**D**) Overlap of downregulated lncRNAs in HIV and TNF. (**E**,**F**) Differential expression of TALAM1 measured by RNA-seq (**E**) and validated by RT–qPCR in HIV-infected C20 (**F**). (**G**,**H**) Differential expression of LINC00702 measured by RNA-seq (**G**) and validated by RT–qPCR in HIV-infected C20 (**H**). Data represent mean ± s.d. (*n* = 4 biological replicates). Data are shown as mean ± s.d.; Student’s *t*-test, *n* = 4, ns ≥ 0.05, *p* < 0.05.

To distinguish virus-specific RNA changes from those produced by TNF-α stimulation independently of infection, we first compared differentially expressed protein-coding transcripts in HIV-1-infected and TNF-α-stimulated cells with those in Mock cells. One well-described consequence of HIV-1 infection in microglia is the induction of proinflammatory cytokines such as TNF-α [8]. Secreted TNF-α can act in an autocrine manner on microglia [40], thereby amplifying inflammatory responses and contributing to the transcriptional changes observed during infection. For this reason, we analyzed HIV-1- and TNF-α-induced transcriptomes separately, to discriminate virus-specific effects from those attributable to cytokine-mediated stimulation. So, we first performed a Venn analysis of differentially expressed protein-coding polyadenylated RNAs. Among the upregulated transcripts, 143 were shared, whereas 183 were unique to HIV-1-infected microglia and 80 were exclusive to TNF-α stimulation (Figure 2A). For downregulated transcripts, 94 were shared, 254 were specific to HIV-1 infection, and 31 were specific to TNF-α stimulation (Figure 2B). This analysis also indicated a substantial overlap between the two conditions, with shared transcripts detected among both upregulated and downregulated genes. More than half of the transcripts differentially expressed under TNF-α stimulation were also detected in the HIV-1-infected condition (64% of the upregulated and 75% of the downregulated transcripts).

Although protein-coding transcripts constitute the majority of differentially expressed RNAs in our dataset, it is equally important to consider lncRNAs, which have emerged as key regulators of cellular and viral processes. HIV-1 infection has been shown to modulate the expression of lncRNAs, particularly in macrophages and T cells, where several have been implicated in controlling distinct stages of the viral replication cycle [21]. Likewise, stimulation with TNF-α alters lncRNA expression, contributing to the fine-tuning of inflammatory responses [30,41]. Despite growing interest in this field, the number of lncRNAs characterized in the context of HIV-1 and virus-triggered neuroinflammation remains limited. Therefore, we investigated the changes in lncRNA expression in HIV-1-infected and TNF-α-stimulated microglia to identify novel candidates potentially involved in viral replication and inflammatory responses. Consistent with coding transcripts, Venn diagrams indicated both shared and condition-specific regulation of lncRNAs (Figure 2C,D). Among the upregulated lncRNAs, 3 were specific to HIV-1-infected microglia, 1 was exclusive to TNF-α stimulation, and 3 were shared. For downregulated lncRNAs, 17 were specific to HIV-1 infection, 4 were exclusive to TNF-α stimulation, and 5 were common to both. A few of the detected lncRNAs correspond to genes annotated as pseudogenes (e.g., *CASTOR3P*, *ENSG00000291132*, *AHSA2P, TMEM198B,* and *DPY19L2P2*); however, the isoforms expressed in our dataset are classified as polyadenylated lncRNAs (see Supplementary Table S2). Analysis of these transcripts revealed distinct expression profiles: some were exclusively regulated by HIV-1, others by TNF-α, and a subset was co-regulated under both conditions (Table 1). Most HIV-1-exclusive lncRNAs remain uncharacterized, with no previous functional annotation or reported biological role associated with viral infection, inflammatory signaling, or other biological processes. Therefore, this group represents the largest fraction of potentially novel lncRNAs identified in this study. In contrast, the TNF-α–exclusive lncRNAs were mostly previously described, either in cancer or in association with viral or inflammatory responses, with only one transcript lacking prior characterization. The group of shared lncRNAs (HIV-1/TNF-α) included both well-known transcripts, such as NEAT1 and CDKN2B-AS1, already implicated in HIV-1 infection, and others previously associated with different viral contexts, including SARS-CoV-2, enterovirus D68, and parainfluenza virus.

Because our primary aim was to identify lncRNAs directly associated with HIV-1 infection in microglia, either through direct viral regulation or through HIV-1–induced inflammatory pathways, we focused subsequent analyses on comparing HIV-1-infected and Mock cells, using TNF-α stimulation only as a reference to distinguish virus-specific from general inflammatory responses. We specifically focused on upregulated lncRNAs, as their induction during HIV infection suggests active involvement in regulatory processes triggered by the virus. Therefore, from the three upregulated lncRNAs exclusively induced by HIV-1, we selected TALAM1 (the most strongly upregulated and exclusively induced by HIV-1) and LINC00702 (the most upregulated lncRNA among those also responsive to TNF-α) for further validation. TALAM1 expression was significantly increased in HIV-1-infected microglia compared to Mock controls, as observed both in RNA-seq (log_2_(fold change) ≈ 1.3; Figure 2E) and RT-qPCR (log_2_(fold change) ≈ 0.9; Figure 2F). Similarly, LINC00702 showed a consistent upregulation in infected cells, with comparable trends in RNA-seq (log_2_(fold change) ≈ 0.7; Figure 2G) and RT-qPCR validation confirming a significant increase (log_2_(fold change) ≈ 0.5; Figure 2H). To explore the temporal pattern of TALAM1 and LINC00702 expression following HIV-1 infection, we additionally evaluated their levels at 6 and 48 h post-infection by RT-qPCR (Supplementary Figure S3). The results showed that TALAM1 expression increased at 6 h post-infection, followed by a decreasing trend at 48 h. In contrast, LINC00702 did not exhibit significant changes at either 6 or 48 h post-infection, although a slight upward trend was observed. These observations suggest that the transcriptional modulation of the lncRNAs TALAM1 and LINC00702 detected at 24 h is driven by HIV-1 infection and, in the case of LINC00702, may be linked to post-transcriptional stages of the provirus. These findings identify TALAM1 as an HIV-1-responsive lncRNA in microglia and confirm the reliability of the transcriptomic data. The RT-qPCR results further validate the RNA-seq observations, demonstrating a consistent upregulation of both TALAM1 and LINC00702 in HIV-1-infected cells. Together, these data highlight the potential involvement of these lncRNAs in the microglial response to HIV-1 infection, providing a foundation for future mechanistic studies.

### 2.3. Silencing of TALAM1 and LINC00702

**Figure 3:**
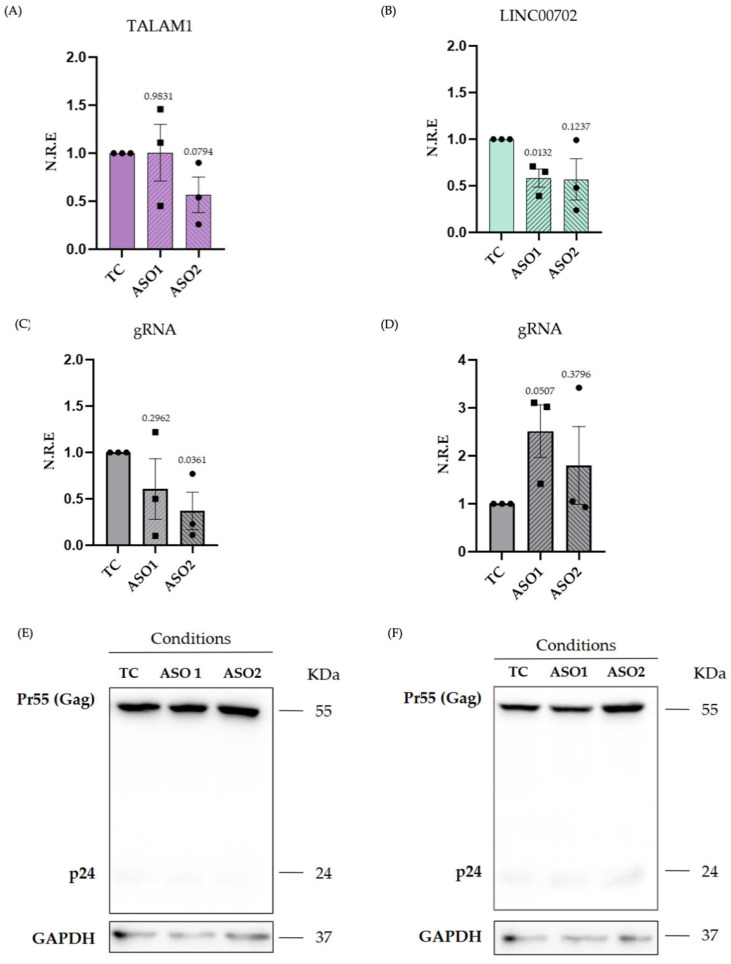
Effect of TALAM1 and LINC0702 silencing on HIV-1 infection in C20 microglia. (**A**,**B**) Relative expression of TALAM1 (**A**) and LINC00702 (**B**) in HIV-infected C20 cells after silencing with two independent antisense oligonucleotides (ASOs), compared with transfection control (TC). (**C**,**D**) Relative HIV-1 genomic RNA (gRNA) levels following TALAM1 (**C**) or LINC00702 (**D**) silencing. (**E**,**F**) HIV-1 protein expression after TALAM1 (**E**) or LINC00702 (**F**) silencing, showing Gag p55 (55 kDa) and p24 (24 kDa), with GAPDH (37 kDa) as loading control. Data are shown as mean ± s.d.; Unpaired *t*-test, *n* = 3, ns ≥ 0.05, *p* < 0.05.

To explore the potential functional contribution of TALAM1 and LINC00702 during HIV-1 infection, we performed knockdown experiments using two independent antisense oligonucleotides (ASOs) targeting each transcript in C20 cells infected with HIV-1. RT-qPCR analysis confirmed a reduction in TALAM1 and LINC00702 expression following ASO treatment (Figure 3A,B). For TALAM1, ASO1 produced minimal reduction in transcript levels, whereas ASO2 resulted in an approximately 43.3% decrease, although this effect did not reach statistical significance due to variability between replicates. On the other hand, LINC00702 expression was reduced by ~43% with ASO1 and ~41.6% with ASO2; however, only ASO1-mediated knockdown reached statistical significance. Assessment of viral genomic RNA (gRNA) revealed a significant reduction in viral RNA levels upon TALAM1 knockdown, particularly evident with ASO2 (Figure 3C). In contrast, LINC00702 silencing increased viral RNA levels, with ASO1 showing a stronger trend toward increased gRNA compared with ASO2, although variability between replicates was observed (Figure 3D). We also evaluated Pr55(Gag) levels under these conditions. While TALAM1 silencing did not affect Pr55(Gag) protein levels (Figure 3E), LINC00702 silencing resulted in increased Pr55(Gag) expression (Figure 3F). Together, these observations suggest that TALAM1 and LINC00702 exert distinct effects during HIV-1 infection. TALAM1 depletion is associated with reduced viral RNA levels without detectable changes in Gag protein production, whereas LINC00702 silencing increases both viral RNA abundance and Pr55(Gag) expression, consistent with a potential negative regulatory role in viral replication.

### 2.4. Subcellular Localization of TALAM1 and LINC00702 Is Differentially Affected by HIV-1 Infection

**Figure 4:**
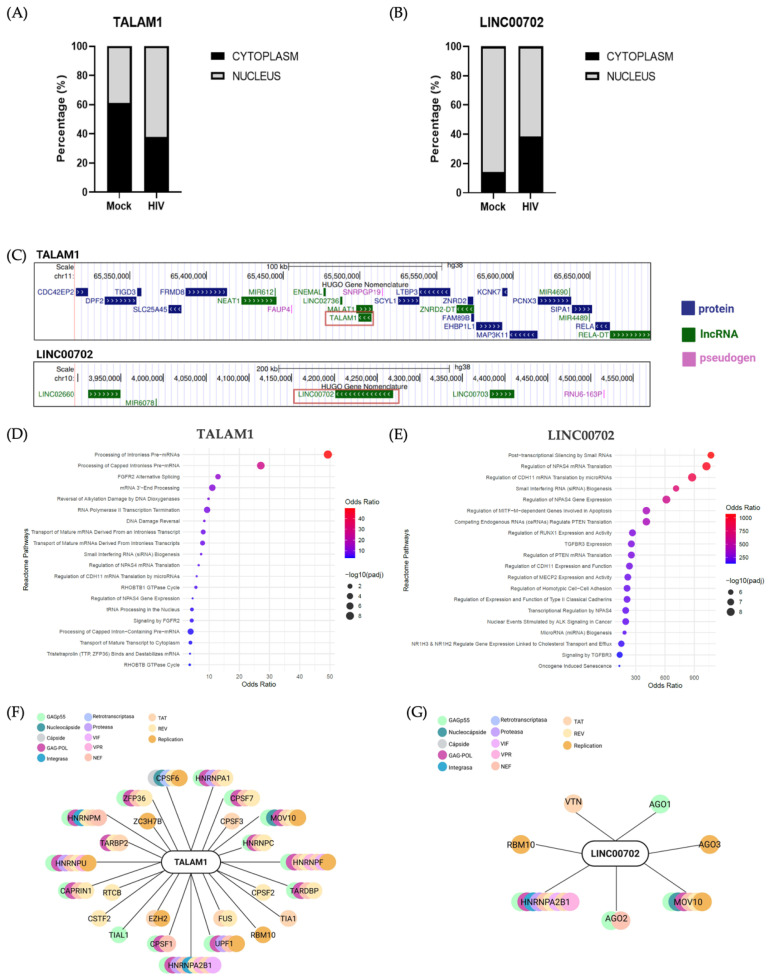
Functional characterization of TALAM1 and LINC00702 interactomes. (**A**,**B**) Subcellular distribution of TALAM1 (**A**) and LINC00702 (**B**) in nuclear and cytoplasmic fractions from Mock and HIV-1 infected C20 microglial cells at 24 h post-infection. Values represent the relative percentage of RNA detected in each compartment. Data are shown as mean ± s.d., Two-way ANOVA, *n* = 3, ns ≥ 0.05, *p* < 0.05. (**C**) Genomic neighborhood of TALAM1 and LINC00702 visualized using the UCSC Genome Browser. (**D**) Reactome pathways enriched for TALAM1 target genes predicted by NPInter v5.0. (**E**) Reactome pathways enriched for LINC00702 target genes predicted by NPInter v5.0. (**F**,**G**) Overlap between HIV-1-associated proteins (HIV-1 Human Interaction Database) and predicted target genes of TALAM1 and LINC00702.

Because the subcellular localization of lncRNAs is often indicative of their functional mechanisms, we next investigated whether HIV-1 infection influences the nuclear–cytoplasmic distribution of TALAM1 and LINC00702. The efficiency of cytoplasmic and nuclear fractionation was validated by Western blot analysis of compartment-specific markers (Supplementary Figure S4). The relative localization of TALAM1 and LINC00702 between cytoplasmic and nuclear compartments was then assessed under Mock and HIV conditions (Figure 4A,B). TALAM1 displayed a predominantly cytoplasmic localization in Mock cells, whereas HIV-1 infection was associated with an increased nuclear fraction and a corresponding reduction in the cytoplasmic proportion. Two-way ANOVA revealed a significant interaction between infection status and subcellular localization, indicating that HIV-1 infection alters the nuclear–cytoplasmic balance of TALAM1. In contrast, LINC00702 exhibited strong nuclear enrichment under both conditions. A significant effect of subcellular localization confirmed its predominantly nuclear distribution. Although HIV-1 infection did not produce a statistically significant interaction effect, a modest increase in cytoplasmic representation was observed that did not reach statistical significance. Together, these results indicate that TALAM1 undergoes an HIV-1-associated shift toward the nuclear compartment, whereas LINC00702 remains predominantly nuclear regardless of infection status.

### 2.5. Genomic Context and Predicted Interaction Networks of TALAM1 and LINC00702

To elucidate the potential function of the selected lncRNAs, we applied two complementary approaches. Given that lncRNAs may act either in *cis* by influencing nearby genes or in *trans* by modulating distant targets, we first analyzed their genomic context. TALAM1 corresponds to the MALAT1 antisense lncRNA. It is located near the well-characterized lncRNA NEAT1, a key component of nuclear paraspeckles [77] (Figure 4C, up). Both MALAT1 and NEAT1 have already been characterized during HIV-1 infection [24,32]. Within a 100 kb window, TALAM1 is also surrounded by several protein-coding genes, including FRMD8, linked to inflammation [78]; SCYL1, involved in neuronal function and, together with SCYL2, in HIV-1 regulation [79,80]; SLC25A42, a regulator of HIV-1 transcription via Tat [81]; LTBP3, an immune-related gene potentially regulated by lncRNAs and associated with TGF-β signaling during HIV-1 infection [82]; DPF2, a chromatin adaptor that connects the SWI/SNF complex with the NF-κB pathway and promotes its activation during inflammatory responses [83] and MAP3K11, associated with antiviral responses [84], among others (Figure 4C, up). In contrast, LINC00702, located on chromosome 10, required the analysis of a broader 200 kb window due to its intergenic nature. This region contains no protein-coding genes and comprises only a few non-coding elements, including LINC02660, MIR6078, LINC00703, and the pseudogene RNU6-163P (Figure 4C, bottom). Among them, only LINC00703 has been reported as a tumor suppressor in gastric cancer [85], while no studies have yet described functional roles for the others (Figure 4C, bottom).

As a complementary approach, we investigated potential interaction partners of TALAM1 and LINC00702 using the NPInter database, which compiles experimentally supported and computationally predicted interactions of non-coding RNAs with proteins, microRNAs (miRNAs), and other non-coding RNAs [86]. While this strategy provides valuable insights into molecules that may physically associate with the selected lncRNAs, it is limited by excluding downstream regulatory targets. The full list of predicted interactions is provided in Supplementary Table S3. For LINC00702, interactions were directly retrieved under its official name. TALAM1 was absent under its standard annotation, but matching interactions were detected under the alternative ID NONHSAG008671, as annotated in GeneCards. We next performed pathway enrichment analysis of TALAM1 and LINC00702-associated proteins obtained from NPInter v5.0 using the Reactome tool from EnrichR. Because the reported lncRNA–protein interactions in this database are derived from curated literature and computational predictions, they should be interpreted as putative associations rather than experimentally validated interactions in our system. Interestingly, TALAM1 interactors were significantly enriched in RNA splicing and mRNA processing pathways, consistent with its genomic proximity to NEAT1 and its potential role in nuclear RNA metabolism (Figure 4D). On the other hand, LINC00702-associated proteins were enriched in translation-related pathways (Figure 4E). Both RNA processing and translational control are key cellular processes targeted by HIV-1 and other viruses [87,88], supporting the notion that TALAM1 and LINC00702 may modulate complementary stages of viral gene expression. Finally, to explore their potential involvement in HIV-1 infection, we cross-referenced the predicted protein interactors with the HIV-1 Human Interaction Database. TALAM1 was found to interact with 25 cellular proteins that directly associate with viral proteins, most notably with Rev, Gag-Pol, and Pr55(Gag) (Figure 4F). In comparison, LINC00702 displayed a more restricted interactome, with seven cellular proteins linked to viral replication, primarily interacting with Pr55(Gag) (Figure 4G). Despite this smaller network, several LINC00702-associated proteins are involved in immune and antiviral responses, suggesting a potential immunomodulatory role for this lncRNA. For instance, hnRNPA2B1 triggers innate immune signaling and enhances IFN-α/β production in response to DNA viruses [89]; MOV10 regulates interferon signaling and exerts antiviral activity against multiple RNA viruses [90]; and vitronectin (VTN) is associated with neuroinflammatory and neurodegenerative processes [91]. Additionally, in viruses such as dengue, RBM10 can regulate the inflammatory response through RIG-I or modulate inflammatory signaling through the RIG-I pathway, and interacts with NF-κB–regulatory lncRNAs during viral infection [92,93], while Argonaute proteins (AGO1, AGO2, and AGO3) contribute to interferon-mediated antiviral defense by controlling virus-derived RNA and modulating immune signaling [94,95,96]. Collectively, these findings suggest that TALAM1 and LINC00702 may contribute to distinct yet complementary aspects of HIV-1 infection in microglia. TALAM1 appears to be primarily involved in nuclear RNA metabolism and splicing regulation, consistent with its proximity to NEAT1 and its extensive interactome with virus-associated proteins. In contrast, LINC00702 interacts with a smaller set of proteins, but several of them have established roles in immune and antiviral responses, suggesting a potential function in modulating microglial inflammatory and antiviral pathways. Together, these observations highlight TALAM1 and LINC00702 as microglial lncRNAs with complementary regulatory roles, providing a basis for future studies exploring their contributions to viral replication and neuroinflammation. Together, these analyses suggest that TALAM1 and LINC00702 may participate in distinct regulatory processes during HIV-1 infection in microglia. The HIV-1-associated nuclear redistribution of TALAM1, combined with the enrichment of its predicted interactors in RNA processing and splicing pathways, is consistent with a potential role in nuclear RNA metabolism. In contrast, LINC00702 remains predominantly nuclear and is associated with proteins involved in translational control and antiviral responses, suggesting that it may be linked to immune-related regulatory pathways. Further functional studies will be required to determine how these lncRNAs influence microglial responses during HIV-1 infection.

## 3. Discussion

HIV-1 persistence in the CNS remains a major barrier to viral eradication, with microglia acting as the principal reservoir in the brain and key drivers of chronic neuroinflammation [2]. LncRNAs have emerged as crucial regulators of gene expression during infection and immune activation; however, their involvement in HIV-1–microglia interactions remain poorly understood. In this study, we investigated the effects of HIV-1 on microglial transcriptional responses, distinguishing infection-associated changes from those linked to virus-induced inflammation mediated by TNF-α, in order to identify lncRNAs with potential regulatory functions. Transcriptomic analyses were performed at 24 h post infection, a time point widely used in HIV-1 models in which viral integration and productive infection have already been established [36,97,98]. Functional enrichment analysis of differentially expressed protein-coding genes in HIV-1-infected and TNF-α-stimulated microglia revealed distinct transcriptomic signatures that share overlapping features of viral and inflammatory responses (Figure 1D). These findings validate the relevance of our *in vitro* model for studying HIV-1-driven transcriptional alterations in the CNS. Notably, the number of genes modulated by HIV-1 was higher than that induced by TNF-α alone, suggesting a broader and more complex transcriptional program during viral infection (Figure 2A–D), likely reflecting the complexity of viral infection. Whereas TNF-α represents a defined inflammatory stimulus, HIV-1 simultaneously activates multiple cellular pathways, including antiviral sensing, transcriptional reprogramming, and stress-associated signaling, resulting in a broader transcriptional landscape in infected microglia.

Considering the intricate and context-dependent nature of cytokine regulation, recent studies have underscored the role of lncRNAs in modulating interleukin expression and other immune-related pathways in various pathological contexts [99,100]. This prompted us to investigate whether the lncRNAs altered in HIV-1-infected microglia were linked to virus-specific or inflammation-associated transcriptional programs. Among the ~30 differentially expressed lncRNAs identified in HIV-1-infected microglia, several were associated with immune and transcriptional regulatory processes (Table 1). Within this group, TALAM1 and LINC00702 stood out as the most consistently regulated transcripts across conditions. TALAM1 was exclusively upregulated in HIV-1-infected microglia, whereas LINC00702 was upregulated under both HIV-1 and TNF-α stimulation, pointing to distinct but potentially convergent regulatory functions. RT-qPCR analyses confirmed the consistent upregulation of both lncRNAs, reinforcing the robustness and reproducibility of our transcriptomic data (Figure 2F,H). Functional silencing experiments indicate that both TALAM1 and LINC00702 contribute to the HIV-1 infection process, as their depletion differentially affected viral replication: TALAM1 knockdown was associated with a reduction in viral gRNA levels, whereas LINC00702 knockdown resulted in increased gRNA and Gag p55 protein levels (Figure 3). Although the precise molecular mechanisms underlying these effects remain unclear, accumulating evidence indicates that lncRNAs frequently participate in host–virus interactions by regulating transcription, RNA processing, or translation of viral and host transcripts. For example, silencing of lncRNA-BTX in peripheral macrophages significantly reduced vesicular stomatitis virus and Sendai virus RNA levels, highlighting a role for lncRNAs in antiviral innate responses [101]. Similarly, LINC02574 has been shown to inhibit influenza A virus replication by promoting the expression of interferon-related genes [102], while RNA interference-mediated downregulation of the lncRNA U90926 markedly suppressed HSV-1 DNA replication and viral proliferation [103]. Together, these observations illustrate the capacity of lncRNAs to influence viral infection through diverse regulatory mechanisms. In our case, the subcellular fractionation analysis provides preliminary clues regarding the potential functions of these lncRNAs (Figure 4A,B). Additional studies are required to determine their precise roles, but the relative distribution of TALAM1 and LINC00702 between nuclear and cytoplasmic compartments is consistent with the functional profiles suggested by their predicted protein interaction networks and also with the effect that reducing the levels of these lncRNAs has on viral RNA and protein. In particular, TALAM1-associated proteins are mainly involved in RNA processing and transcriptional regulation, processes typically linked to nuclear functions, whereas the interaction network of LINC00702 is enriched for proteins related to translational regulation, which can be correlated by the observed increase in Pr55(Gag) protein levels following LINC00702 silencing (Figure 3F). Although these interactions were inferred from publicly available datasets and have not yet been experimentally validated in microglial models, the concordance between subcellular localization and predicted molecular partners provides a useful framework to guide future mechanistic studies aimed at defining the roles of these lncRNAs during HIV-1 infection. In line with these observations, TALAM1 and LINC00702 present features that may be relevant to host–virus interactions. TALAM1, the antisense transcript of MALAT1, is known to stabilize its sense partner, which participates in nuclear organization and RNA processing [104,105]. In our model, TALAM1 was identified as a transcript specifically inducible by HIV-1 infection. Its genomic context includes protein-coding genes previously linked to HIV-1 infection and inflammation, such as DPF2, LTBP3, and MAP3K11, while its predicted protein interactors participate in RNA metabolism, splicing, and transcriptional regulation. Moreover, several TALAM1-associated proteins directly interact with HIV-1 components, including Rev, Gag-Pol, and Pr55(Gag), reinforcing its potential role in viral RNA processing and gene expression. Notably, TALAM1 has also been reported to be upregulated in other viral infections, including enteroviruses and SARS-CoV-2, although its function in these contexts remains largely unexplored [42,43]. By contrast, LINC00702, which was regulated by both HIV-1 and TNF-α in our dataset, displays an interaction network enriched for proteins involved in translational processes and immune-related pathways. This functional profile includes proteins previously associated with antiviral and inflammatory responses, which may help explain its coordinated upregulation under both stimuli. Although the study is limited to a single time point, focuses only on polyadenylated RNAs, and relies on unvalidated bioinformatic predictions, the RT-qPCR validation expands the repertoire of lncRNAs associated with the microglial response to HIV-1 and provides a framework for future mechanistic studies on their role in CNS viral pathogenesis.

## 4. Materials and Methods

### 4.1. Cell Culture

The C20 human microglial [39] and HEK293T (human embryonic kidney 293 with SV40 T-antigen) cell lines were maintained in Dulbecco’s Modified Eagle Medium DMEM (HyClone™, Cytiva, Marlborough, MA, USA) supplemented with 10% FBS (HyClone™, Cytiva, Marlborough, MA, USA) and 1% Pen-Strep-Ampho B solution (Biological Industries, ThermoFisher Scientific, Waltham, MA, USA) at 37 °C and 5% CO_2_ atmosphere.

### 4.2. HIV-1 Pseudotyped VSVg Production

In total, 3 × 10^6^ HEK293T cells were cultured in 150 mm dishes and were transfected with 5 μg of pCMV-VSVg [106] and 5 μg of pNL4.3ΔEnv proviral vector [107], which abolished Env expression, using lipofectamine^®^ 3000 Transfection kit (Invitrogen, Waltham, MA, USA), diluted in medium without serum Opti-MEM (Gibco, ThermoFisher Scientific, Waltham, MA, USA). At 72 h post-transfection, the pseudotyped virus was recovered from the supernatants, filtered through a 0.22 μm filter, and the p24 viral protein was quantified using the HIV-1 Gag p24 Quantikine ELISA Kit (R&D Systems, Minneapolis, MN, USA) according to the manufacturer’s instructions, as described previously [108].

### 4.3. Infection of C20 Cells

For RNA-seq experiments, 5 × 10^6^ C20 cells were grown in 150 mm plates. After 24 h, C20 cells were infected with previously prepared VSVg-pseudotyped HIV-1 at 250 ng/mL of p24. After two hours, the culture medium was removed, and the cells were washed twice with PBS 1X (HyClone^TM^, Cytiva, Marlborough, MA, USA). Fresh DMEM medium supplemented with 10% FBS (HyClone™) was added, and cells were incubated at 37 °C for 24 h. As a positive control for microglial activation, we stimulated microglia with recombinant TNF-α at 10 pg/mL (Gibco, ThermoFisher Scientific, Waltham, MA, USA), as described in Garcia-Mesa et al. (2017) [39]. Uninfected cells were maintained as a control (Mock). For RT-qPCR validation of lncRNA expression, experiments were performed at a reduced scale using 2 × 10^5^ cells per condition cultured in 6-well plates. Cells were harvested 24 h post-infection, corresponding to the primary time point analyzed in this study. Additionally, 6 h and 48 h post-infection were included to evaluate early and late transcriptional responses.

### 4.4. Cell Viability Assay

Cell viability of C20 microglial cells under mock, HIV-1 infection, or TNF-α stimulation conditions was assessed by measuring LDH release using the Pierce LDH Cytotoxicity Assay Kit (ThermoFisher Scientific, Waltham, MA, USA) according to the manufacturer’s instructions with minor modifications. C20 cells were subjected to the same experimental conditions described in Section 4.3. After 8 h post-infection, cells were trypsinized and seeded in 96-well plates at a density of 4.5 × 10^3^ cells per well in 100 μL DMEM supplemented with 1% FBS to minimize serum background. LDH release was measured 24 h post-infection. Maximum LDH release controls were generated by adding 20 μL of lysis buffer and incubating for 30 min at 37 °C. An additional maximum LDH release control was included for HIV-1-infected cells. Subsequently, 100 μL of LDH reaction mixture was added to each well and incubated for 30 min at room temperature, protected from light. The reaction was stopped by adding 50 μL of stop solution, and absorbance was measured at 490 nm using an Infinite M200 Pro Microplate Reader (Tecan, Männedorf, Switzerland).

### 4.5. RNA Extraction and RT-qPCR

RNA extraction and RT-qPCR were performed as described above [108]. Briefly, C20 cells were washed and recovered in PBS 1X using scrapers. Cells in PBS (HyClone™) were centrifuged at 3000× *g* for 10 min at 4 °C. Samples were resuspended in 200 μL of PBS 1X, and the RNA was extracted using 1 mL of TRIzol™ (Invitrogen, ThermoFisher Scientific, Waltham, MA, USA) and 200 μL of chloroform (Merck, Darmstadt, Germany) according to the manufacturer’s instructions. The mix was vortexed, incubated for 5 min at room temperature, and centrifuged at 12,000× *g* for 15 min at 4 °C. The aqueous phase was recovered and incubated for 5 min with an equal volume of isopropanol (Merck, Darmstadt, Germany). The mix was vortexed and centrifuged at 12,000× *g* for 10 min at 4 °C. The RNA pellet obtained was washed with 500 μL of ETOH 70% (Merck, Darmstadt, Germany) and resuspended in ultrapure water (Corning, NY, USA). The RNAs obtained were treated with RQ1 DNase (Promega, Madison, WI, USA) according to the manufacturer’s instructions. Reverse transcription (RT-PCR) was performed using 1000 ng of RNA and the High-Capacity cDNA Reverse Transcription Kit with RNase Inhibitor (Applied Biosystems, ThermoFisher Scientific, Waltham, MA, USA) as indicated by the manufacturer (just samples for lncRNAs validation). A no-RT control was performed, adding RNA but not the RT enzyme. The cDNA obtained was used to perform qPCR with Brilliant II SYBR^®^ Green QPCR Master Mix (Agilent Technologies, Santa Clara, CA, USA) as described previously [108]. Primers used are indicated in Supplementary Table S4. The amplification was performed using the AriaMx Real-Time PCR System (Agilent Technologies, Santa Clara, CA, USA). GAPDH, a housekeeping gene, was used as a control reference. A no-cDNA negative control using water was included. Relative copy numbers of interest RNA were normalized to GAPDH using 2^ΔΔCt^ [109].

### 4.6. Western Blot

To validate HIV-1 infection, cellular pellets from Mock, TNF-α-stimulated, and HIV-1-infected microglia were resuspended in RIPA buffer (Cell Signaling, Danvers, MA, USA, #5871S). Protein concentrations were determined using the Pierce BCA Protein Assay Kit (ThermoFisher Scientific, Waltham, MA, USA) and samples were then heated at 95 °C for 10 min. For Western blotting, 25 µg of total protein per sample were loaded onto a 12% SDS–PAGE gel and electrophoresed at 80 V for 30 min, followed by 120 V for 2 h. Proteins were transferred onto a nitrocellulose membrane (Bio-Rad, Hercules, CA, USA) at 100 V for 2 h. Membranes were blocked with a 5% (*w*/*v*) blocking solution (Bio-Rad, Hercules, CA, USA) for 1 h at room temperature, then washed twice with PBS containing 0.1% (*v*/*v*) Tween-20 (PBS-T). Subsequently, membranes were incubated overnight at 4 °C with the primary antibody against HIV-1 p24 (mouse monoclonal, 1:3000; NIH AIDS Reagent Program, #3537), lamin A (mouse monoclonal, 1:1000, Cell Signaling, Danvers, MA, USA, #86846) or α-tubulin (mouse monoclonal, 1:1000, Cell Signaling, Danvers, MA, USA, #3873). GAPDH was used as a loading control and detected with a mouse monoclonal anti-GAPDH antibody conjugated to HRP (1:1000; Cell Signaling, Danvers, MA, USA, #51332S). Membranes incubated with the p24 antibody were washed three times with PBS-T and then incubated with HRP-conjugated mouse IgG secondary antibody (1:5000; Jackson ImmunoResearch, West Grove, PA, USA) for 2 h at room temperature. Protein bands were visualized using the Immobilon Forte Western HRP Substrate (Merck, Darmstadt, Germany) for 30 s to 2 min and imaged with the UVITEC Cambridge FireReader^®^ system (UVITEC, Cambridge, UK).

### 4.7. RNA-Seq

RNA extraction from C20 cells: Mock, infected with HIV-1, or stimulated with TNF-α was performed from cell extracts as described above. For the preparation of Poly(A)+ RNA, an mRNA isolation kit (Roche, Basel, Switzerland) was used. 50 µg of total RNA was diluted in 100 µL of ultrapure water (Corning, NY, USA) and 100 µL of Lysis Buffer, then incubated at 65 °C for 2 min. 1.5 µL of OligodT marked with biotin was added and mixed. Finally, all the lysis buffer was removed. Samples were resuspended in a hybridization mix (Sample + OligodT) and incubated at 37 °C for 5 min. Samples were separated from the liquid using a magnetic rack for 3 min. Subsequently, samples were resuspended in 250 µL of Wash Buffer and separated using the magnetic rack. To elute the RNA, samples were resuspended using 25 µL of ultrapure water (Corning, NY, USA) and incubated at 65 °C for 2 min. Then, samples were separated from the eluate using magnetic shock. The supernatant was saved in an RNase-free tube and quantified. ~50 µg of RNA was treated using RNA Fragmentation Reagents (ThermoFisher Scientific, Waltham, MA, USA); RNA was resuspended in 18 µL of Nuclease-free Water. Then, 2 μL of the 10X Fragmentation Buffer was added, mixed, spun briefly, and incubated at 70 °C for 15 min in a heating block. Finally, 2 μL of the Stop Solution was added and stored at −80 °C. cDNA library preparation and RNA-seq were performed as a service from Genoma Mayor at Universidad Mayor, Chile. All the samples were sequenced on an Illumina HiSeq2000 platform with paired-end 100 to 150 bp read length. RNA-seq data availability. Raw sequencing data were submitted to the National Center for Biotechnology Information (NCBI) under Submission ID SUB15424263, associated with BioProject PRJNA1289834. As the BioProject is currently under private status during peer review, sequencing files can be accessed through the reviewer link: https://dataview.ncbi.nlm.nih.gov/object/PRJNA1289834?reviewer=ai0am0pikc73a2ut5kt6e3dh82 (accessed on 2 December 2025). Only datasets corresponding to non-IP samples were included in the present analysis.

### 4.8. RNA-Seq Data Processing and Expression Analysis

Sequenced reads were quality checked on FastQC. To ensure robust differential expression analysis, reads were mapped using Salmon [110] and aligned using STAR [111]. Transcript-level quantification was performed using raw RNA-seq reads against the GENCODE v44 human transcriptome reference. Salmon was executed multiple times with different settings to optimize mapping efficiency and quantification accuracy. Quantification results from Salmon were imported into R using the Tximeta package [112], which allowed for the incorporation of transcript-level metadata, including technical replicates and bootstrap information. Differential expression was assessed at both gene and transcript levels using complementary methods. For gene-level analysis, differential expression was calculated using DESeq2 [113]. For transcript-level analysis, we employed swish, a method within the fishpond package [114], which is specifically designed to integrate bootstrap-derived technical replicates and is optimized for transcript-level inference. Only genes or transcripts with an adjusted *p*-adjust < 0.05 and absolute Log2[fold change] > 0.5 were considered differentially expressed. These thresholds were chosen based on literature and empirical observation that lncRNAs and transcript isoforms typically exhibit lower expression levels than protein-coding genes [115], and higher fold change cutoffs (e.g., 1 or 2) would result in substantial loss of potentially relevant signals. A final list of differentially expressed genes (DEGs) and differentially expressed transcripts (DETs) was generated by selecting consensus hits across methods, thus ensuring reliable detection of expression changes.

### 4.9. Antisense Oligonucleotide (ASO)-Mediated lncRNA Silencing

To silence both lncRNAs, antisense oligonucleotides (ASOs) were designed to selectively deplete human TALAM1 and LINC00702 using an RNase H–dependent gapmer strategy. All ASOs contained a fully phosphorothioated backbone (Supplementary Table S5). For TALAM1, gapmers with 2′-O-methoxyethyl (2′-MOE) modifications in the 5′ and 3′ flanking regions were designed to target a unique region not overlapping with the MALAT1 sense transcript. For LINC00702, gapmers incorporating Affinity Plus (LNA-like) sugar modifications in the flanking regions were designed to target a sequence within the terminal exon shared by most annotated isoforms. Two independent ASOs per target were selected based on GC content and predicted hybridization properties. ASO sequences are provided in Supplementary Table S5. C20 cells were transfected with ASOs (10 nM) using Lipofectamine RNAiMAX (ThermoFisher Scientific, Waltham, MA, USA) according to the manufacturer’s recommendations. C20 cells were maintained in DMEM supplemented with 1% FBS without antibiotics during transfection. After 6 h, the medium was replaced with DMEM containing 1% FBS and antibiotics, and cells were further incubated under standard culture conditions. Medium plus Opti-MEM and Lipofectamine RNAiMAX without ASO was used as a transfection control.

### 4.10. Subcellular Fractionation 

To assess the subcellular localization of TALAM1 and LINC00702, cytoplasmic and nuclear fractions were isolated from C20 microglial cells as previously described [36]. Briefly, C20 cells were collected 24 h post-infection and were washed twice with ice-cold PBS 1X and centrifuged at 500× *g* for 5 min at 4 °C. An aliquot (300 μL) was reserved as input. For cytoplasmic and nuclear separation, cell pellets were resuspended in 300 μL of lysis buffer 1 (10 mM HEPES, 10 mM NaCl, 3 mM CaCl_2_, 0.1% Nonidet-P40, 1X protease inhibitor cocktail), gently mixed five times, and centrifuged at 10,000× *g* for 30 s at 4 °C. The supernatant was collected as the cytoplasmic fraction. The pellet was washed once with lysis buffer 1 and subsequently resuspended in lysis buffer 2 (1 M NaCl, 1 M Tris-HCl pH 7.5, 0.5% Nonidet-P40, 1 mM EDTA, 1X protease inhibitor cocktail) to obtain the nuclear fraction. Both fractions were centrifuged at 16,000× *g* for 5 min at 4 °C, and supernatants were collected for RNA extraction and protein quantification. Relative RNA levels were determined by RT-qPCR using the comparative Ct method (ΔCt), normalizing each transcript to GAPDH as an internal control. For each experimental condition (Mock or HIV-1 infection), cytoplasmic and nuclear values were calculated independently and subsequently normalized so that the sum of both compartments equaled 100%. These normalized values were used to determine the percentage distribution of each transcript between cytoplasmic and nuclear compartments, which were represented as 100% stacked bar plots. Statistical analysis was performed using ordinary two-way ANOVA, with experimental condition (Mock versus HIV-1) and subcellular localization (cytoplasm versus nucleus) as independent variables. Šídák’s multiple comparisons test was applied where appropriate. Statistical significance was set at *p* < 0.05.

### 4.11. Prediction of lncRNA-Interacting Biomolecules

To investigate potential interactions between selected lncRNAs and cellular biomolecules, we queried NPInter v5.0, a curated database of RNA-biomolecule associations primarily derived from high-throughput experiments (http://bigdata.ibp.ac.cn/npinter5/, accessed on 20 January 2025). The list of biomolecules was then filtered against a list of interactions between cellular proteins and viral proteins of HIV-1 [116].

### 4.12. Functional Enrichment Analysis

To identify biological processes associated with the differentially expressed protein-coding genes in HIV-1-infected and TNF-α-stimulated microglia, a Fast Gene Set Enrichment Analysis (fgsea) was performed. The fgsea algorithm provides a fast and statistically robust estimation of enrichment by permuting gene ranks rather than sample labels [117]. Enrichment scores were expressed as normalized enrichment scores (NES), while the −log_10_(adjusted *p*-value) was used to indicate significance and represented by dot size in visualization plots. For the enrichment analysis of the total interactome associated with each lncRNA, protein–RNA interaction data were retrieved from NPInter v5.0. The list of interacting proteins was then analyzed using the Reactome Pathway module implemented in EnrichR to identify overrepresented biological pathways. The enrichment results were visualized as dot plots, where color represents the odds ratio and dot size corresponds to −log_10_(adjusted *p*-value), providing an integrated view of the magnitude and significance of pathway enrichment.

## 5. Conclusions

In this study, we characterized the polyadenylated transcriptome of human microglial cells upon HIV-1 infection and TNF-α stimulation, identifying TALAM1 and LINC00702 as consistently regulated lncRNAs. Functional analyses revealed that these lncRNAs play distinct roles in the HIV-1 infection, with TALAM1 associated with the regulation of viral RNA levels and LINC00702 acting as a potential negative regulator of viral replication. Their differential subcellular localization and redistribution upon infection further support distinct mechanistic roles. Likewise, their genomic context and predicted interactions suggest potential involvement in RNA metabolism, translational regulation, and antiviral or inflammatory responses. While these findings are based on an in vitro model and bioinformatic predictions, they provide a valuable framework for future functional studies to elucidate the roles of TALAM1 and LINC00702 in HIV-1 biology within the central nervous system. Overall, this work expands the catalogue of non-coding transcripts associated with microglial responses to HIV-1 and contributes to a better understanding of lncRNA-mediated mechanisms in neurovirology.

## Figures and Tables

**Table 1:** Differentially expressed lncRNAs in microglia infected with HIV-1 or stimulated with TNF-α.

Condition	Name	Description	Biological Context Reported	
			Virus	Others
HIV-1 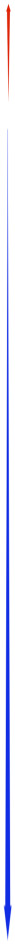	TALAM1 (NONHSAG008671)	TALAM1 transcript, MALAT1 antisense RNA	1. ↑ in Enterovirus D68 infection [42] 2. ↑ in COVID patients [43]	1. ↑ in primary lung cancer [44]
	ENSG00000268287	Novel transcript	1.↑in SARS-CoV-2 [45]	Not described yet
	ENSG00000293339	Novel transcript	Not described yet	Not described yet
	ASMTL-AS1	ASMTL antisense RNA 1	1. ↓ in Human parainfluenza virus 3 [46] 2. ↓ in SARS-CoV-2 infection [46]	1. ↑ in tumor tissue from colorectal cancer [47]
	LINC02593	Long intergenic non-protein coding RNA 2593	Not described yet	1. ↓ in pancreatic cancer [48]
	CDKN2B-AS1	CDKN2B and CDKN2A antisense cis and trans regulatory RNA 1	1. ↑ in Enterovirus 71 infection [49] 2. ↓ in HIV-1 infection [32]	1. ↑ in various cancers [50]
	ZNF710-AS1	ZNF710 antisense RNA 1	Not described yet	1. ↑ in gastric cancer cell [51]
	CASTOR3P *	CASTOR family member 3, pseudogene	Not described yet	Not described yet
	ENSG00000291132 *	myotubularin related protein 9 like, pseudogene	Not described yet	1. Biomarker in multiple myeloma [52]
	AHSA2P *	activator of HSP90 ATPase homolog 2, pseudogene	Not described yet	Not described yet
	LINC00639	long intergenic non-protein coding RNA 639	1.↓ in SARS-CoV-2 infection [46]	1. ↓ in HIV-associated dementia (HAD) [53]
	TMEM198B *	transmembrane protein 198B (pseudogene)	Not described yet	Not described yet
	ENSG00000293413	novel transcript	Not described yet	Not described yet
	ENSG00000272668	novel transcript, antisense to VSIG8	Not described yet	Not described yet
	PSMB8-AS1	PSMB8 antisense RNA 1 (head to head)	1. ↑ in Influenza A virus infection with different strains [54]	1. ↑ in human atherosclerotic plaques [55] 2. ↑ in pancreatic cancer [56]
	PIK3CD-AS2	PIK3CD antisense RNA 2	1. ↑ in H5N1 infection [57] 2. ↑ in SARS-CoV-2 infection [46]	Not described yet
	MIAT	myocardial infarction associated transcript	Not described yet	1. ↑ in lung cancer [58]
	MELTF-AS1	MELTF antisense RNA 1	1. ↑ in Enterovirus 71 infection [49] 2. ↓ in Zika virus infection [59]	1. ↑ in osteosarcoma tissues [60]
	ENSG00000260293	novel transcript, intronic to TBC1D24	Not described yet	1. ↑ in lung adenocarcinoma tumor tissues [61]
	EOLA1-DT	EOLA1 divergent transcript	Not described yet	1. ↓ in thyroid carcinoma [62] 2. ↓ in colon cancer [63]
HIV-1/TNF-α 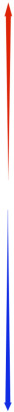	LINC00702	Long intergenic non-protein coding RNA 702	Not described yet	1. ↑ in malignant meningioma tissues [64] 2. ↓ in non-small cell lung cancer [65]
	ENSG00000280800	novel transcript, similar to YY1 associated myogenesis RNA 1 YAM1	Not described yet	Not described yet
	ENSG00000268108	novel transcript, antisense to NTF4	1. ↑ in SARS-CoV-2 infection [45]	Not described yet
	NEAT1	nuclear paraspeckle assembly transcript 1	1. ↑ in HIV-1 infection [32] 2. ↑ in SARS-CoV-2 infection [46] 3. ↑ in HSV-1 infection [66]	1. ↑ in tumor tissues and hepatoma cells [67] 2. Upregulated in colorectal cancer [68]
	ZNF436-AS1	ZNF436 antisense RNA 1	Not described yet	1. ↑ in human calcific aortic valve tissues [69]
	HCP5	HLA complex P5	1. ↑ in Human parainfluenza virus 3 [46] 2. ↑ in SARS-CoV-2 infection [46]	1. ↑ in gastric cancer [70]
	LINC00963	long intergenic non-protein coding RNA 963	Not described yet	1. ↑ in different types of cancer [71]
	DPY19L2P2 *	DPY19L2 pseudogene 2	1. ↑ in Enterovirus 71 infection [49]	Not described yet
TNF-α 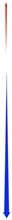	LOXL1-AS1	LOXL1 antisense RNA 1	1. ↑ in SARS-CoV-2 infection [46]	1. ↑ in hepatocellular carcinoma [72]
	SCOC-AS1	SCOC antisense RNA 1	Not described yet	1. Biomarker in salt sensitivity of blood pressure [73]
	ENSG00000287160	novel transcript	Not described yet	Not described yet
	LINC01551	Long intergenic non-protein coding RNA 1551	1. ↑ in Enterovirus 71 infection [49]	1. ↑ in nasopharyngeal carcinoma [74]
	OIP5-AS1	OIP5 antisense RNA 1	1. ↑ in Coxsackie virus B3 infection [75] 2. ↑ in SARS-CoV-2 infection [46]	1. Associated with different cancers [76]

* Isoform found corresponds to lncRNA; ↑ upregulated lncRNA; ↓ downregulated lncRNA; ↑ = reported as upregulated; ↓ reported as downregulated.

## Data Availability

The raw RNA-seq data generated in this study have been deposited in the NCBI Sequence Read Archive under Submission ID SUB15424263, BioProject PRJNA1289834. During peer review, data are accessible through the reviewer link: https://dataview.ncbi.nlm.nih.gov/object/PRJNA1289834?reviewer=ai0am0pikc73a2ut5kt6e3dh82. (accessed on 2 December 2025).

## Supplementary Materials

The following supporting information can be downloaded at: https://www.mdpi.com/article/10.3390/ijms27073271/s1.
