# Supplementary material for: Transcriptomic Profiling Identifies TALAM1 and LINC00702 as HIV-1-Responsive lncRNAs in Microglia
Source: Int J Mol Sci. 2026 Apr 4;27(7):3271. doi: 10.3390/ijms27073271 (PMC13073277; doi:10.3390/ijms27073271)
Supplement: Supplementary file 1 [file ijms-27-03271-s001.zip › Supplementary Figures S1-S4.pdf]

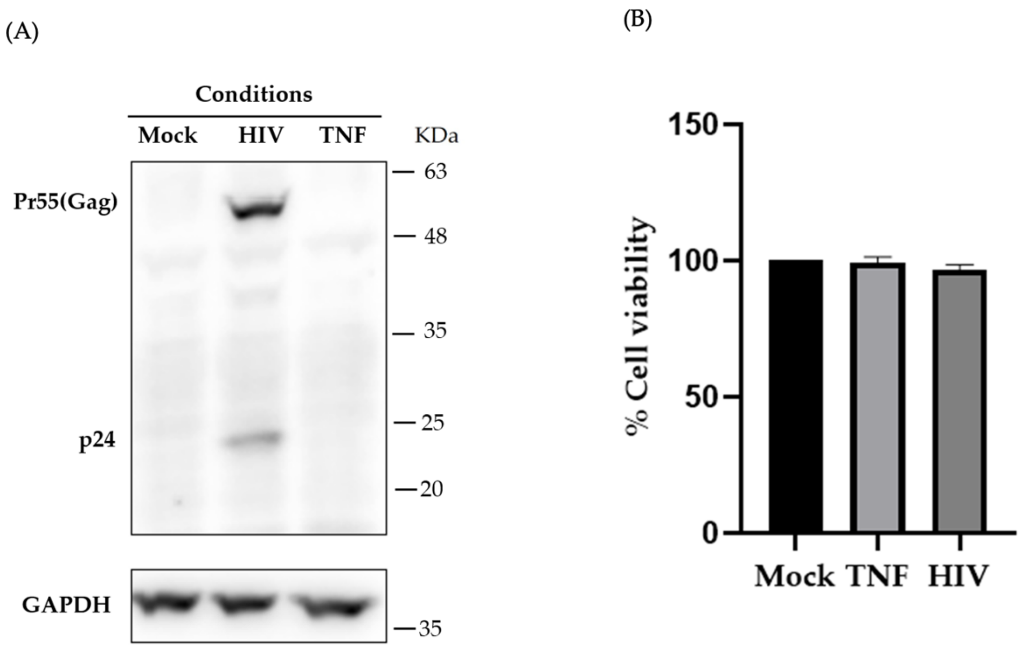

Supplementary Figure S1. Efficient HIV-1 infection of C20 cells with no detectable impact on cell viability. (**A**) Representative Western blot showing the levels of the viral proteins Pr55(Gag) and p24 in Mock-, HIV-1–infected, and TNF-α–treated C20 microglial cells. GAPDH was used as a loading control. (**B**) Cell viability assessed by lactate dehydrogenase (LDH) released in C20 microglial cells under the indicated conditions. Data are presented as percentage of viable cells. No significant differences were observed between treatments (n = 3 biological replicates).

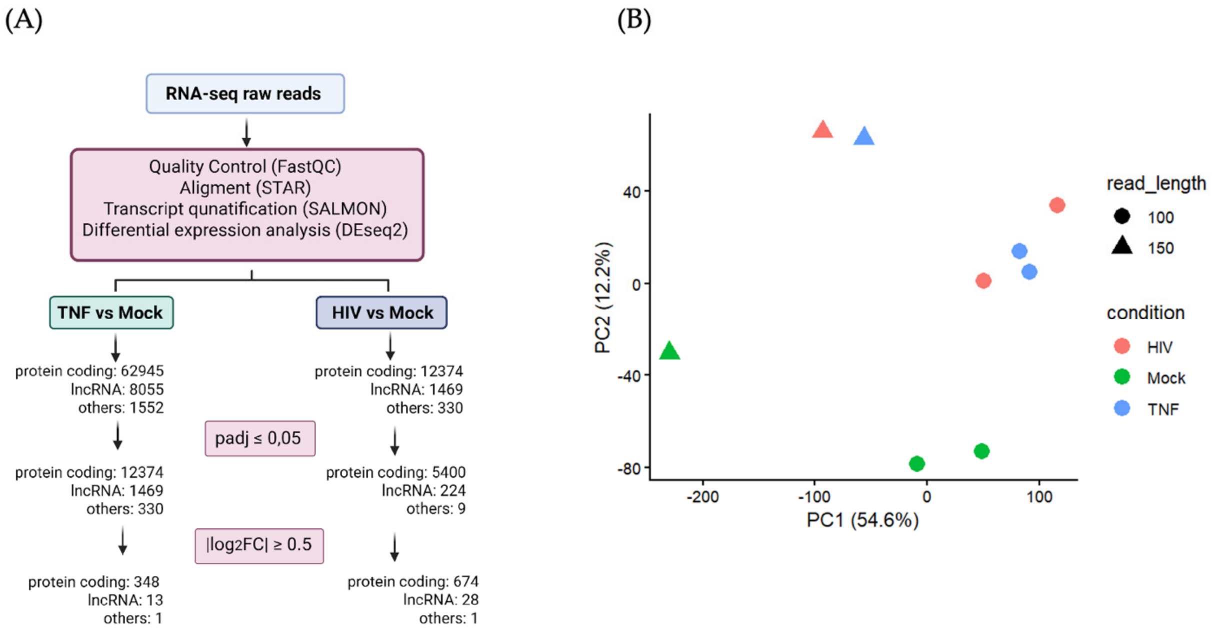

Supplementary Figure S2. RNA-seq analysis overview and sample clustering. (**A**) Workflow of the RNA-seq analysis pipeline used for the HIV vs Mock and TNF vs Mock comparisons. Transcript counts obtained after alignment and quantification were analyzed using DESeq2. Statistical significance was determined using FDR-adjusted p-values, and transcripts with adjusted p-value < 0.05 were retained and further filtered by $|\log_2 FC| \geq 0.5$. The number of transcript types remaining after each filtering step is indicated. (**B**) Principal component analysis (PCA) of normalized transcript

counts showing separation of samples according to experimental condition (Mock, TNF, and HIV). PC1 explains 54.6% of the total variance. Samples are additionally annotated by sequencing read length (100 bp and 150 bp), indicating that experimental condition rather than read length represents the main source of transcriptomic variation.

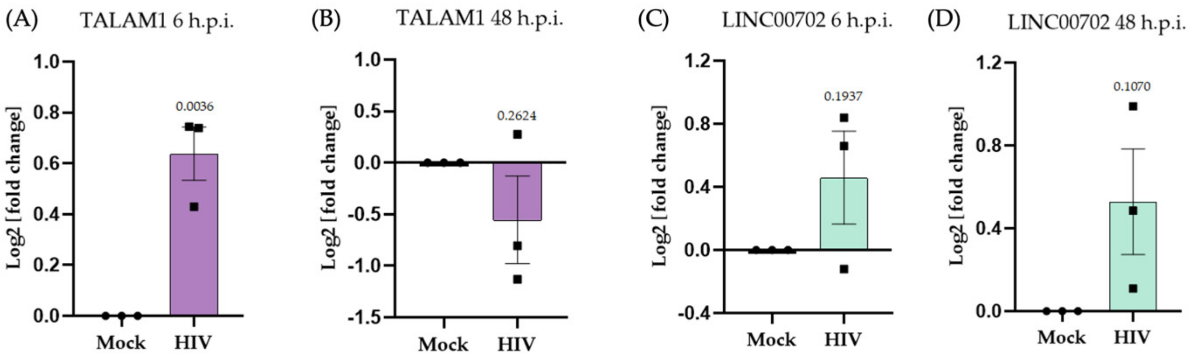

Supplementary Figure S3. Temporal expression of TALAM1 and LINC00702 following HIV-1 infection. Relative expression levels of the lncRNAs TALAM1 and LINC00702 were measured at early and late time points after HIV-1 infection measured by RT-qPCR. (**A**) TALAM1 expression at 6 hours post-infection and Mock. (**B**) TALAM1 expression at 48 hours post-infection and Mock. (**C**) LINC00702 expression at 6 hours post-infection and Mock. (**D**) LINC00702 expression at 48 hours post-infection and Mock. Data are shown as mean ± s.d.; Student's *t*-test, $n = 3$, ns ≥ 0.05, $p < 0.05$.

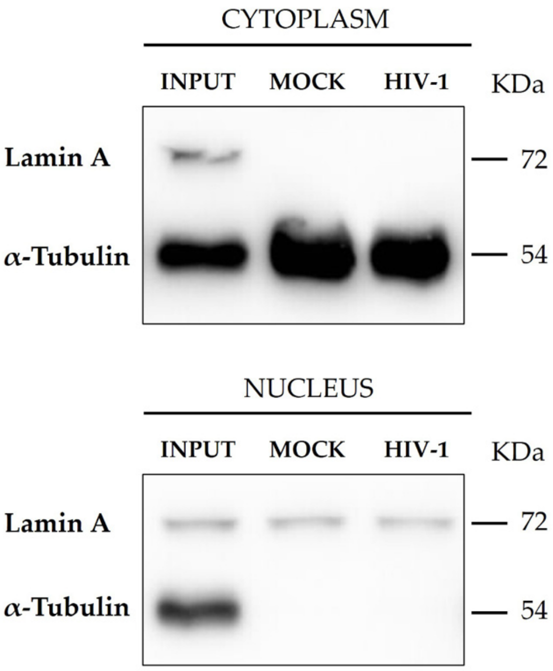

Supplementary Figure S4. Validation of nuclear and cytoplasmic fractionation. Representative Western blot showing enrichment of Lamin A (nuclear marker) and α-Tubulin (cytoplasmic marker) in the corresponding fractions. Whole-cell lysate (input) and nuclear and cytoplasmic fractions from Mock and HIV-1 infected C20 microglial cells at 24 h post-infection are shown.
