# Supplementary material for: Transcriptomic Profiling Identifies TALAM1 and LINC00702 as HIV-1-Responsive lncRNAs in Microglia
Source: Int J Mol Sci. 2026 Apr 4;27(7):3271. doi: 10.3390/ijms27073271 (PMC13073277; doi:10.3390/ijms27073271)
Supplement: Supplementary file 1 [file ijms-27-03271-s001.zip › Supplementary Tables S1,S2,S4,S5.pdf]

Supplementary Table S1. Differential expression (Log_2FC) of selected genes associated with TNF/NF-κB signaling and inflammatory cytokine responses in HIV-1–infected and TNF-α–stimulated microglia.

Gene Name	Log_2FC HIVvsMock	Log_2FC TNFvsMock
NFKB1	0.790	0.671
RELB	1.634	0.936
IRAK1	0.657	0.618
IL6	4.393	2.065
CXCL8	4.649	0.743

Supplementary Table S2. Differentially expressed transcripts annotated as lncRNAs in our dataset, identified using Ensembl BioMart (GRCh38.p14).

Condition	GeneID	Rowname	Gen Symbol	Annotation
**HIV-1**	ENSG00000289740	ENSG00000289740.1	TALAM1	lncRNA
	ENSG00000268287	ENSG00000268287.1	ENSG00000268287	lncRNA
	ENSG00000293339	ENSG00000293339.1	ENSG00000293339	lncRNA
	ENSG00000236017	ENSG00000236017.8	ASMTL-AS1	lncRNA
	ENSG00000223764	ENSG00000223764.2	LINC02593	lncRNA
	ENSG00000259291	ENSG00000259291.2	ZNF710-AS1	lncRNA
	ENSG00000291122	ENSG00000291122.1	CASTOR3P	annotated pseudogene, expressed isoform classified as lncRNA
	ENSG00000291132	ENSG00000291132.1	ENSG00000291132	annotated pseudogene, expressed isoform classified as lncRNA
	ENSG00000293434	ENSG00000293434.1	AHSA2P	annotated pseudogene, expressed isoform classified as lncRNA
	ENSG00000259070	ENSG00000259070.9	LINC00639	lncRNA
	ENSG00000293491	ENSG00000293491.1	TMEM198B	annotated pseudogene, expressed isoform classified as lncRNA
	ENSG00000293413	ENSG00000293413.1	ENSG00000293413	lncRNA
	ENSG00000272668	ENSG00000272668.2	ENSG00000272668	lncRNA
	ENSG00000204261	ENSG00000204261.9	PSMB8-AS1	lncRNA
	ENSG00000231789	ENSG00000231789.4	PIK3CD-AS2	lncRNA
	ENSG00000225783	ENSG00000225783.9	MIAT	lncRNA
	ENSG00000228109	ENSG00000228109.2	MELTF-AS1	lncRNA
	ENSG00000260293	ENSG00000260293.2	ENSG00000260293	lncRNA
	ENSG00000241769	ENSG00000241769.7	EOLA1-DT	lncRNA
**HIV-1 /TNF-α**	ENSG00000233117	ENSG00000233117.4	LINC00702	lncRNA
	ENSG00000280800	ENSG00000280800.1	ENSG00000280800	lncRNA
	ENSG00000268108	ENSG00000268108.1	ENSG00000268108	lncRNA
	ENSG00000245532	ENSG00000245532.11	NEAT1	lncRNA
	ENSG00000249087	ENSG00000249087.9	ZNF436-AS1	lncRNA
	ENSG00000206337	ENSG00000206337.12	HCP5	lncRNA
	ENSG00000204054	ENSG00000204054.14	LINC00963	lncRNA
	ENSG00000291139	ENSG00000291139.1	DPY19L2P2	annotated pseudogene, expressed isoform classified as lncRNA
**TNF-α**	ENSG00000261801	ENSG00000261801.8	LOXL1-AS1	lncRNA
	ENSG00000196951	ENSG00000196951.13	SCOC-AS1	lncRNA
	ENSG00000287160	ENSG00000287160.1	ENSG00000287160	lncRNA
	ENSG00000186960	ENSG00000186960.14	LINC01551	lncRNA
	ENSG00000247556	ENSG00000247556.7	OIP5-AS1	lncRNA

Supplementary Table S4. Pairs of primers used in this study.

Primer	Forward (5´-3´)	Reverse (5´-3´)
genomic RNA HIV-1	AGCGAAAGTAAAGCCAGAGG	TCTCTCTCCTTCTAGCCTCC
TALAM1	GTTCCCCAGGCTGGAAGATT	GGTGAAATACCCTCCCTGGC
LINC00702	AGACGAAGTGCTCCTGATGG	TCCATGTGAACACACGCTGA
GAPDH	AGCCACATCGCTCAGACAC	GCCCAATACGACCAAATCC

Supplementary Table S5. Antisense oligonucleotide (ASO) used in this study.

LncRNA	ASO1	ASO2
TALAM1	CGTCTGAAGAATCTTCCAGC	AGTAAACCCAGACCTACCAG
LINC00702	GCATTCACGTTGGAATCTCC	TGACTGCTAAGATGATTGAA
